# Biological and Technological Characteristics of Milk-Fermented with Probiotic *Lactobacillus acidophilus* La-5 and Viable/Inactivated *Saccharomyces boulardii* 002Y018 Cultures

**DOI:** 10.1007/s12602-026-10920-3

**Published:** 2026-02-10

**Authors:** Hany Elkashef, Hoda M. Elzeini, Islam M. Shawky, Ashwak Abel Moneim Hassan

**Affiliations:** Dairy department, Faculty of Agriculture, Cairo University, PO Box 12613, Giza, Egypt

**Keywords:** Fermented milk, Saccharomyces, Lactobacillus, Biological characteristics

## Abstract

This study was design to evaluate the effect of using of probiotic *Saccharomyces boulardii* 002Y018 strain in the viable or inactivated form on the technological and biological properties of milk-fermented with probiotic *Lactobacillus acidophilus* La-5 culture. The results demonstrated that the addition of *S. boulardii* 002Y018 strain in the viable form negatively affected the growth of *L. acidophilus* La-5, but in its inactivated form, it enhanced the viable count of *L. acidophilus* La-5 compared to that of milk-fermented with *L. acidophilus* La-5 as a single culture. At the beginning or after 21 days of cold storage, the viable counts of *L. acidophilus* La-5 or *S. boulardii* 002Y018 culture in all fermented milk treatments still meet the recommended minimum requirements of 6 Log CFU/mL for probiotic strains. Milk-fermented with *S. boulardii* 002Y018 culture in its different forms recorded the highest scores of organoleptic attributes. Milk-fermented with co-culture of probiotic strains exhibited the greatest values of proteolysis, ACE-I, and antioxidant abilities on day 1 or after 21 days of storage period. All fermented milk treatments showed a great inhibitory effect on the growth of some pathogenic bacteria and fungi. Fermentation of milk with *L. acidophilus* La-5 and *S. boulardii* 002Y018 as a co-culture improved the production of organics acids. Also, the addition of *S. boulardii* 002Y018 strain promoted the ability to inhibit α-amylase and α-glucosidase that is related to the reduction of diabetes. Ultimately, our findings provide that *S. boulardii* 002Y018 strain can be successfully applied, in the viable or inactivated form, for the manufacturing of functional fermented milk rich in bioactive components.

## Introduction

The contemporary lifestyle, prevailed by unhealthy of people mentality, nutrition, and body physical, has led to various health disorders or diseases including malnutrition, obesity, cardiovascular, diabetes, and mental health. Several previous studies have carried out to produce probiotic-fortified food products that are designated to present health effects beyond essential nutrition, herewith serving to the growing health-awareness consumer market [1]. Recently, because of the above reasons and the increasing of consumer awareness with the health nutrition, the food and dairy industries have realized the necessity to control or prevent these issues through the production of functional food products by their enriching with probiotic microorganisms that possess several positive effects on the human health [2].

Since old eras, probiotic microorganisms have performed a crucial role in the manufacturing of functional fermented food products. It should take into consideration that not all fermented food products domesticate to act as probiotic carriers since some processes may inhibit or eliminate the probiotic cultures [3]. However, milk and dairy products are considered a perfect environment for the fortification with probiotics. Probiotic microorganisms are live, non-pathogens, which accord positive health effects to the human body when consumed in appropriate quantities [4]. Regarding probiotic bacteria, *Bifidobacterium*, *Lactobacillus*, *Lactococcus*, and *Streptococcus* genera are the most predominant probiotics [5]. However, a novel edge is emerging in the commune of probiotics-the elevation of probiotic yeasts. Among the fully explored probiotic yeasts, *Candida milleri*, *Kluyveromyces lactis*, *Debaroyomyces hansenii*,* Yarrowia lipolytica*, and *Saccharomyces boulardii* species have been studied for their prospective probiotic properties and ability to grow in fermented food products [6, 7]. For example, *Saccharomyces boulardii*, recognized for its capability to inhibit pathogenic and spoilage bacteria in the intestine, grows at 20–37 °C and through a pH range of 4–6 [8]. In addition, de Souza et al. [9] mentioned that *Kluyveromyces lactis* participates in the dairy butter manufacturing via generating effective enzymes, which improving product quality and consumer acceptability. Recently, various terms have been suggested for the applying of probiotic cell compounds and metabolites including parabiotics and postbiotics. The term of postbiotics have been indicated to metabolites released post inactivation or inhibition of microorganisms [10].

Recently, the development of functional fermented dairy products rich in bioactive components are the most emerging. One of the natural biocomponents that have received a worthy attention in recent years is bioactive peptides [11]. Peres Fabbri et al. [12] mentioned that bioactive peptides are recognized as unambiguous segments of food proteins containing 2–20 amino acids residues. Purohit et al. [13] illustrated that bioactive peptides can be generated from the main food proteins via the proteolysis by endogenous or exogenous enzymes, food manufacturing procedures, or fermentation by different microorganisms. Despite of proteolysis by enzymes is the utmost popular technique for releasing bioactive peptides from their sources, fermentation is certainly a useful approach to produce bioactive peptides with various health effects because of its low cost and the produced bioactive peptides can be refined without more hydrolysis [14]. When absorbed, bioactive peptides have exhibited diverse biological effects in the human body including antihypertensive, antimicrobial, antidiabetic, anticancer, antioxidant, and immune-enhancement characteristics [15].

The growing realization of the importance of functional foods has promoted the production of bioactive peptides in the fermented dairy products via microbial fermentation. The future of probiotic yeasts utilization in the manufacturing of dairy products possess significant promise, and the scientific research is constantly recognizing innovative approaches to maximize their therapeutic effects and evolve new dairy products. Considering the above, this study was designed to develop novel functional fermented milk rich in bioactive peptides using probiotic *L. acidophilus* La-5, *S. boulardii* 002Y018, and its postbiotic.

## Materials and Methods

### Materials

Fresh cow milk (11.65%total solids, 3.10% protein, and 3.40% fat) was purchased from Dairy Technology Center, Faculty of Agriculture, Cairo University, Egypt. Skim milk powder (SMP) was obtained from Arla Foods Company (Viby J, Denmark).

Direct vat inoculation (DVI) starter of probiotic *Lactobacillus acidophilus* La-5 and *Saccharomyces cerevisiae* var. *boulardii* 002Y018 strain were procured from MIFAD-Misr food additives, Cairo, Egypt, and the Regional Center of Mycology and Biotechnology, Al-Azhar University, Cairo, Egypt, respectively. Helmy et al. [16] confirmed that isolated *S. cerevisiae* var. *boulardii* 002Y018 strain had probiotic characteristics.

All fine chemical agents, de Man Rogosa and Sharpe (MRS) broth, MacConkey agar, and yeast peptone dextrose medium were purchased from Merck (Egyptian Int. Center for Import, Cairo, Egypt).

### Starters’ Preparation

Freeze-dried *L. acidophilus* La-5 culture was pre-activated (0.02%) in sterilized skimmed milk and incubated at 37 °C overnight. *S. boulardii* 002Y018 strain was pre-cultured in sterilized skimmed milk supplemented with 0.2% glucose and incubated at 28 ± 2 °C for 48 h.

The preparation of inactivated *S. boulardii* 002Y018 cells was conducted in accordance with a technique outlined by Elshaghabee et al. [17]. Briefly, *S. boulardii* 002Y018 was cultured and activated in sterilized yeast peptone dextrose broth, thereafter collected using centrifugation to yield approximately 5 × 10^8^ CFU. These pellets were washed twice with PBS (phosphate-buffered saline with 0.1 M phosphate, 0.15 M NaCl, pH 7.2), were re-suspended in normal saline, were centrifuged, and were subsequently inactivated by laboratory heat-treatment at 75 °C for 15 min. In order to confirm the inactivation of the yeast cells, 100 µL of yeast suspension were examined by spreading onto yeast peptone dextrose agar medium. The inactivated yeast cells, exhibiting no observable growth on the cultured medium after 24 h of incubation at 28 ± 2 °C, were utilized in the fermentation experiment.

### Preparation of Fermented Milk

Three diverse treatments of fermented milk were manufactured at the laboratory scale. Cow milk of all treatments was fortified with 2% of skim milk powder, heat-treated for 10 min at 90 °C, and was cooled to 37 ± 2 °C for inoculation. Heat-treated milk was inoculated with 2% of *L. acidophilus* La-5 culture in the first treatment. In the second and third treatments, heat-treated cow milk was inoculated with 2% co-culture of *L. acidophilus* La-5 and *S. boulardii* 002Y018 (at ratio 1:1) and 2% co-culture of *L. acidophilus* La-5 and inactivated *S. boulardii* 002Y018 (at ratio 1:1), respectively. Inoculated milk in all treatments was incubated at 37 ± 2 °C until fermentation and subsequently was stored at 5 ± 1 °C for 21 days.

### Preparation of Water-Soluble Extract (WSE) of Fermented Milk

According to Abd El-Fattah et al. [18], 10 g of fermented milk was mixed with 40 mL of distilled water in screw-cap tube, and then the mixture was homogenized and incubated for 1 h at 40 °C. After that, the homogenate was centrifuged for 30 min at 10,000 *x*g, and the obtained supernatant (WSE) was collected.

### Physico-Chemical Characteristics

The values of titratable acidity, total solids, protein, and fat of cow milk and fermented milk were estimated according to AOAC methods [19]. The values of pH were determined using a digital pH meter (Adwa AD11, Szeged, Hungary).

### Viscosity and Water-Holding Capacity (WHC)

The apparent viscosity of fermented milk treatments was determined using a concentric cylinder Brookfield digital rotational viscometer (Model DV-II +, Brookfield Engineering laboratories Inc., Middleboro, USA) and using UL adaptor and ULA spindle over a shear rate of 12.2/s. Fermented milk was allowed to temperature at 25 °C for 10 min prior to evaluation, and the values of viscosity (centipoise, cp.) were scored. Concerning WHC, the WHC of fermented milk was assessed for different treatments as performed by Abd El-Fattah et al. [18].

### Microbial Examination

The viable counts (Log CFU/mL) of *L. acidophilus* La-5, *S. boulardii* 002Y018, and coliform bacteria were examined in fermented milk on day 1, 7, and after 21 days of cold storage as described by APHA [20] using MRS agar, yeast peptone dextrose agar, and MacConkey agar media incubated at 37, 28, and 37 °C for 48 h, 5 days, and 24 h, respectively.

### Sensory Evaluation

The sensory evaluation of fermented milk treatments was assessed on day 1 and after 21 days of cold storage. Twenty-five participants from the staff members of the dairy department, Faculty of Agriculture, Cairo University, Egypt assessed the fermented milk treatments for flavor, body and texture, appearance, and overall acceptability using a 9-point hedonic scale where one indicated dislike completely, while nine indicated like totally. All participants were aware of standard sensory assessment, and they had access to distilled water to clean their palates prior assessment. Fermented milk treatments were brought out the refrigerator and were coded randomly one h prior to evaluation to acquire room temperature.

### Protein Hydrolysis

The values of protein hydrolysis were determined by reacting of released free amino residues with *O*-phthaldialdehyde (OPA) as performed by Abd El-Fattah et al. [21].

### Angiotensin Converting Enzyme-Inhibitory (ACE-I) and Antioxidant Ability

The ACE-I and DPPH radical scavenging activities of fermented milk treatments were evaluated as described in the method of Abd El Fattah et al. [21].

### Antimicrobial Activity

The ability of WSEs of fermented milk treatments to inhibit the growth of Methicillin-Resistant *Staphylococcus aureus* ATCC 4330, *Salmonella typhimurium* ATCC 14,028, *Klebsiella pneumoniae* ATCC 13,883, *Aspergillus flavus* RCMB 002002, and *Penicillium italicum* RCMB 001018 was examined using agar well diffusion assay [22, 23]. Ketoconazole (100 µg/mL) and gentamycin (4 µg/mL) as standard antibiotics were used for recognition as positive control samples.

### Organic Acids Content

Sterilized skim milk was inoculated with *L. acidophilus* La-5, *S. boulardii* 002Y018, or mix of them at ratio 2%, incubated at 37 °C and 28 °C, respectively for 24 h, and the cell-free extracts were obtained to determine the organic acid content. The organic acid content in the cell-free extract for *L. acidophilus* La-5, *S. boulardii* 002Y018, and a mix of them was determined as described by Hassan et al. [24]. The extract (20 µL) was injected into an Agilent 1200 high performance liquid chromatography (HPLC) system with a Refractive Index Detector and a REFEX 8 μm 8% H Organic Acid Rezex^@^ column (Phenomenex). Five mmol/L of sulfuric acid was utilized as an elution liquid under circumstances (temperature of the column kept at 65 °C and flow rate = 0.6 mL/min). The original standards of organic acids were conducted under the same circumstances. The retention time of peaks of the extracts was compared with those of organic acid standards and they quantified by estimating area down the peaks.

### Antidiabetic Ability

The ability of fermented milk treatments to inhibit α-amylase and α-glucosidase was applied to estimate the antidiabetic capacity according to Ayyash et al. [25]. The concentrations of 1.95, 3.9, 7.81, 15.62, 31.25, 62.5, 125, 250, 500, and 1000 µg/mL were used as a final concentration of WSE of each fermented milk for the calculation of IC_50_. Acarbose substance was utilized as a positive control.

### Statistical Assessment

A randomized complete block designing and analysis of variance (ANOVA) of factorial methods were conducted using Mstat-C program (Michigan State University). All measurements were determined in triplicates, and the findings were demonstrated as the mean ± standard deviation. The least significant difference (LSD) test was applied to compare among the averages of measurements at the probability of ≤ 0.05. Heatmap correlation matrix plot was carried out using R statistical program (version 4.4.2).

## Results

### Physico-Chemical Characteristics

Non-significant differences were observed among all fermented milk treatments that comprised 13.69 ± 0.02% total solids, 3.31 ± 0.01%, and 3.63 ± 0.013% fat.

Regarding titratable acidity, the results in Table 1 indicate that the addition of *S. boulardii* 002Y018 significantly increased the titratable acidity of fermented milk on day 1 or after 21 days of cold storage. The titratable acidity of fermented milk in all treatments significantly increased with progress of storage time. An opposite trend was noted in the pH values of all fermented milk treatments.

**Table 1:** Change in the titratable acidity, pH, viscosity, and WHC of fermented milk treatments during 21 days of cold storage period

Treatments	Storage days	Acidity (%)	pH	Viscosity (cp.)	WHC (%)
Treatment 1	1	0.707 ± 0.005^e^	4.637 ± 0.12^a^	491.8 ± 5.50^i^	36.567 ± 0.05^g^
	10	0.743 ± 0.005^d^	4.530 ± 0.21^b^	503.4 ± 5.53^h^	39.733 ± 0.37^f^
	21	0.807 ± 0.005^c^	4.427 ± 0.15^c^	513.5 ± 0.60^g^	40.233 ± 0.05^e^
Treatment 2	1	0.753 ± 0.005^d^	4.530 ± 0.11^b^	549.1 ± 3.013^e^	40.87 ± 0.11^d^
	10	0.823 ± 0.010^bc^	4.417 ± 0.17^cd^	555.4 ± 3.80^b^	42.104 ± 0.13^b^
	21	0.860 ± 0.01^a^	4.340 ± 0.21^de^	546.4 ± 4.58^f^	41.950 ± 0.15^b^
Treatment 3	1	0.743 ± 0.005^d^	4.520 ± 0.23^b^	558.4 ± 0.541^a^	41.333 ± 0.05^c^
	10	0.833 ± 0.005^b^	4.400 ± 0.21^cd^	549.8 ± 0.703^d^	40.900 ± 0.65^cd^
	21	0.877 ± 0.011^a^	4.313 ± 0.21^e^	550.9 ± 0.60^c^	43.44 ± 0.03^a^
LSD		0.01715	0.07671	2.364	0.4635

*WHC* water-holding capacity

Means with different superscript small letters in the same column indicate significant differences

Treatment 1: milk-fermented with *L. acidophilus* culture; treatment 2: milk-fermented with *L. acidophilus* and viable *S. boulardii* cultures; Treatment 3: milk-fermented with *L. acidophilus* and attenuated *S. boulardii* cultures

As shown in Table 1, using of *S. boulardii* 002Y018 as a co-culture significantly increased the viscosity and water-holding capacity (WHC) compared to those of milk-fermented with *L. acidophilus* La-5 as a single culture (treatment 1). The rate of increase in viscosity and WHC was 10.40–11.93 and 10.53–11.53%, respectively. With the progress of storage period, the viscosity of fermented milk in treatment 1 significantly increased, while it significantly decreased in the fermented milk of treatments 2 and 3. However, the values of WHC of fermented milk significantly enhanced in all treatments.

### The Viable Counts of Probiotics

**Fig. 1:**
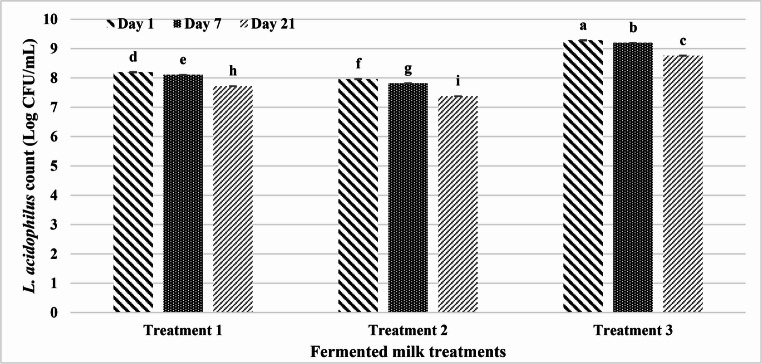
Change in the viable count of *L. acidophilus* La-5 in fermented milk treatments during 21 days of cold storage period. Treatment 1: milk-fermented with *L. acidophilus* culture; treatment 2: milk-fermented with *L. acidophilus* and viable *S. boulardii* cultures; Treatment 3: milk-fermented with *L. acidophilus* and attenuated *S. boulardii* cultures

Fermented milk in all treatments was free from coliform bacteria and count on day 1 and after 21 days of cold storage. The results in Fig. 1 illustrates that using of *S. boulardii* 002Y018 as a co-culture negatively affected the viable counts of *L. acidophilus* La-5 (treatment 2) where the viable counts significantly declined from 8.19 to 7.96 Log CFU/mL. However, using of *S. boulardii* 002Y018 in the inactivated form significantly enhanced the viable counts of *L. acidophilus* La-5 (treatment 3) to 9.28 Log CFU/mL compared to that of treatment 1 (8.19 Log CFU/mL). Our results confirmed that *S. boulardii* 002Y018 grew well in milk and its viable count recorded 7.42 Log CFU/mL in the fermented milk of treatment 2 at the beginning of cold storage, and it significantly decreased to 7.28 Log CFU/mL after 21 days of storage period. In addition, the viable counts of *L. acidophilus* La-5 in all fermented milk treatments significantly declined with the progress of storage time.

### Sensory Characteristics

**Fig. 2:**
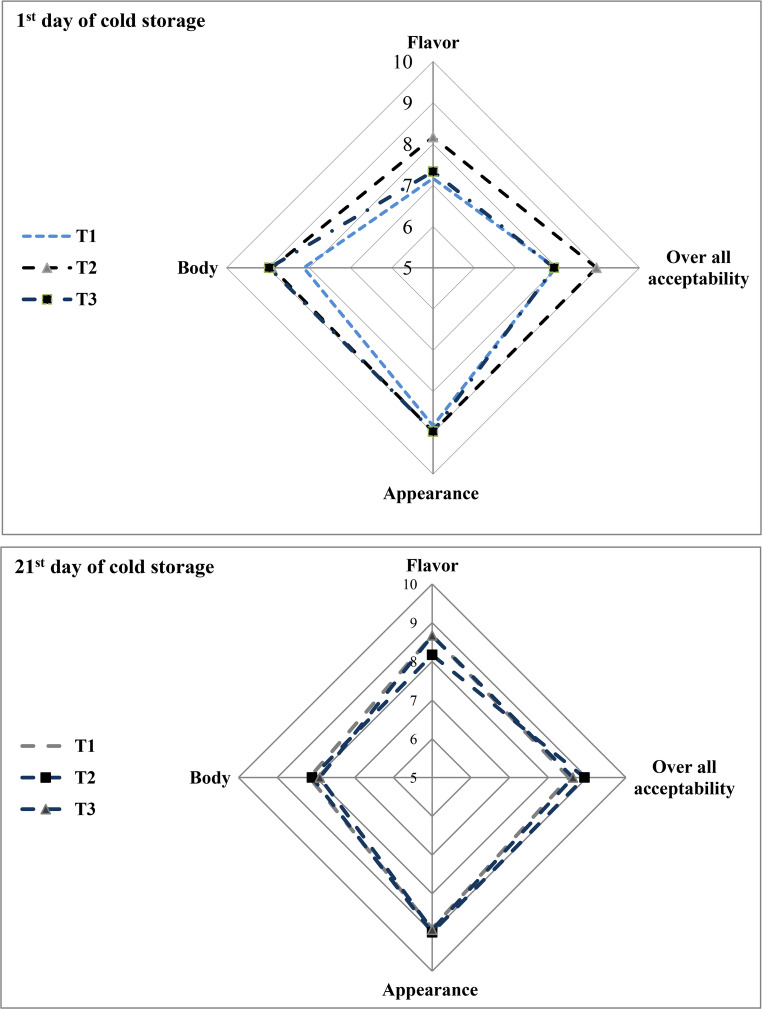
Sensory characteristics of fermented milk treatments on day 1 and after 21 days of cold storage period. Treatment 1: milk-fermented with *L. acidophilus* culture; treatment 2: milk-fermented with *L. acidophilus* and viable *S. boulardii* cultures; Treatment 3: milk-fermented with *L. acidophilus* and attenuated *S. boulardii* cultures

Using probiotic yeasts as a co-culture for the manufacturing of fermented dairy products may affect the organoleptic properties of the product that could cause a reduction in the consumer preference. Hence, this study examined the effect of using a probiotic *S. boulardii* 002Y018 strain on the sensory attributes of fermented milk and their scores are displayed in Fig. 2. On day 1 of cold storage, the addition of *S. boulardii* 002Y018 as a co-culture significantly enhanced the flavor, body and texture, appearance, and overall acceptability of fermented milk (treatments 2) compared to those of the fermented milk in treatment 1. Moreover, using of *S. boulardii* 002Y018 in the inactivated form positively affected the sensory properties of fermented milk (treatment 3). The cold storage period did not negatively affect the score of sensory evaluation of all fermented milk treatments.

### Change in Proteolytic, ACE-I, and Antioxidant Activities

Table 2 shows the variation of proteolytic activity of fermented milk treatments on day 1 and after 21 days of cold storage. On day 1 of or after 21 days of cold storage, milk-fermented with co-culture of *L. acidophilus* La-5 and *S. boulardii* 002Y018 (treatment 2) exhibited the greatest proteolytic activity, followed by milk-fermented with *L. acidophilus* La-5 and inactivated *S. boulardii* 002Y018 (treatment 3). A statistically significant increase was observed in the proteolysis of all fermented milk treatments by 20.38–38.97% along with the prolongation of the storage period.

**Table 2:** Proteolytic, ACE-I, and DPPH radical scavenging activities of fermented milk treatments on day 1 and after 21 days of cold storage period

Treatments	Storage days	Proteolysis (OD_340)_	ACE-I (%)	DPPH radical scavenging (%)
Treatment 1	1	0.371 ± 0.010^e^	44.27 ± 0.67^f^	39.58 ± 3.55^e^
	21	0.466 ± 0.021^d^	53.00 ± 2.06^e^	47.057 ± 1.117^d^
Treatment 2	1	0.632 ± 0.105^c^	70.86 ± 2.044^c^	68.387 ± 3.833^b^
	21	0.932 ± 0.058^a^	88.98 ± 1.016^a^	80.727 ± 3.943^a^
Treatment 3	1	0.484 ± 0.023^d^	61.40 ± 2.022^d^	55.627 ± 3.556^c^
	21	0.793 ± 0.121^b^	77.08 ± 1.637^b^	65.170 ± 3.080^b^
LSD		0.1258	3.168	5.902

*ACE-I* Angiotensin-converting enzyme-inhibitory

Means with different superscript small letters in the same column indicate significant differences

Treatment 1: milk-fermented with *L. acidophilus* culture; treatment 2: milk-fermented with *L. acidophilus* and viable *S. boulardii* cultures; Treatment 3: milk-fermented with *L. acidophilus* and attenuated *S. boulardii* cultures

The ability of WSE of fermented milk treatments to inhibit ACE or scavenge DPPH radical during cold storage is listed in Table 2. The maximum ACE-I and antioxidant activities were noted in milk-fermented with a co-culture of *L. acidophilus* La-5 and *S. boulardii* 002Y018 (treatment 2), while milk-fermented with a single culture of *L. acidophilus* La-5 (treatment 1) had the lowest values after fermentation or at the end of cold storage. Using *S. boulardii* 002Y018 strain in the inactivated form significantly enhanced the ACE-I and antioxidant ability compared to fermented milk of treatment 1. ACE-I and DPPH radical scavenging activities significantly improved by 16.5–20.4% and 14.6–15.3%, respectively in all fermented milk treatments over the cold storage time.

### Antagonistic Activity

**Fig. 3:**
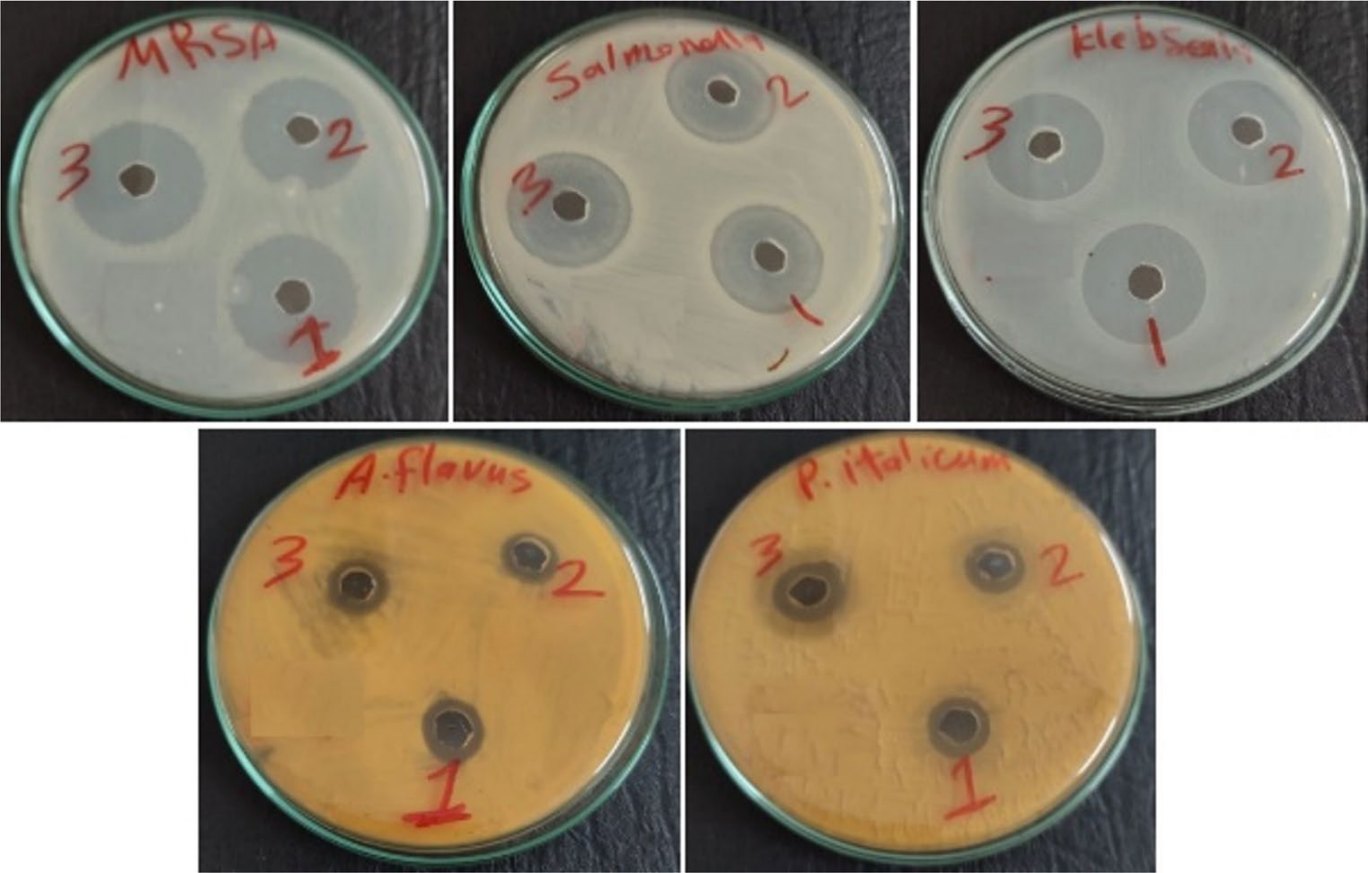
The ability of fermented milk treatments to inhibit *Staph. aureus* MRSA, *Sal. typhmurium*,* Klebsiella pneumonia*,* A. flavus*,* and P. italicum.* Treatment 1: milk-fermented with *L. acidophilus* culture; treatment 2: milk-fermented with *L. acidophilus* and viable *S. boulardii* cultures; Treatment 3: milk-fermented with *L. acidophilus* and attenuated *S. boulardii* cultures

**Fig. 4:**
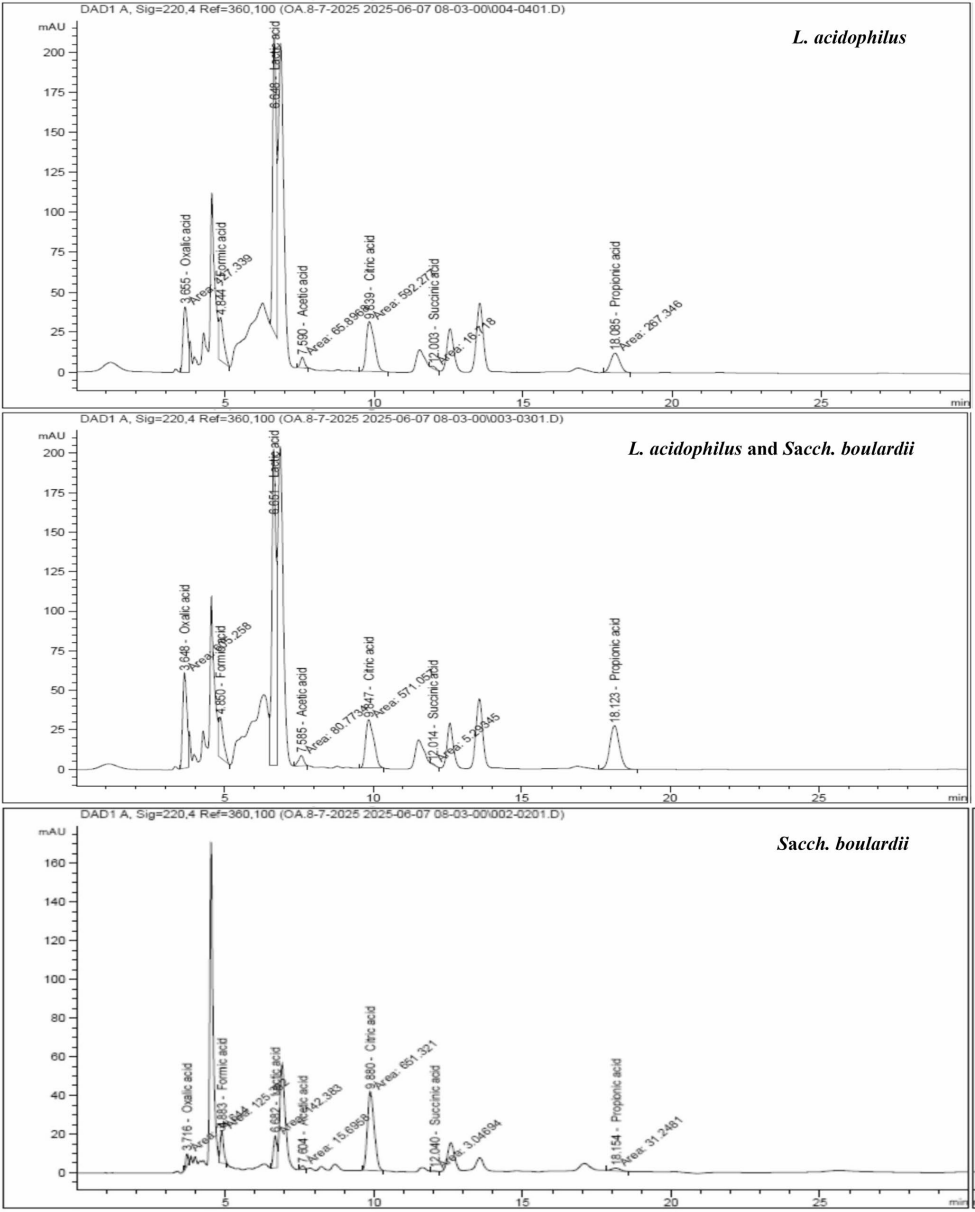
HPLC chromatograms of organic acid profile for *L. acidophilus* as a single culture, *L. acidophilus* and *S. boulardii* as a co-culture, and *S. boulardii* a single culture

The results of antimicrobial effect of fermented milk treatments are presented in Table 3; Fig. 3. All fermented milk treatments exhibited the antimicrobial ability against the growth of three pathogens (MRSA *Staph. aureus* ATCC 4330, *Sal. typhimurium* ATCC 14028, and *Klebsiella pneumoniae* ATCC 13883) and two fungi (*Aspergillus flavus* RCMB 002002 and *Penicillium italicum* RCMB 001018) with different inhibition zone diameters of 11.0–30.0 mm. The addition of *S. boulardii* 002Y018 in the inactivated form led to significantly increase the inhibition zones diameter of fungi and *Staph. aureus* MRSA. As shown in Table 4; Fig. 4, L. *acidophilus* La-5 and/or *S. boulardii* 002Y018 were be able to produce various organic acids in different amounts.

**Table 3:** The antimicrobial activity of fermented milk treatments on the first day of cold storage period

Tested microorganisms	Fermented milk treatments			Control
	Treatment 1	Treatment 2	Treatment 3	
Gram positive bacteria	Zones of inhibition (mm)			Gentamycin
Methicillin-Resistant Staphylococcus aureus ATCC 4330	29.00 ± 0.01^b^	28.00 ± 0.05^c^	30.00 ± 0.00^a^	15.00 ± 0.00
Gram negative bacteria	Zones of inhibition (mm)			Gentamycin
Salmonella typhimurium ATCC 14,028	15.00 ± 0.01^a^	13.99 ± 0.03^b^	13.00 ± 0.01^bc^	17.00 ± 0.00
Klebsiella pneumoniae ATCC 13,883	22.00 ± 0.02^b^	23.00 ± 0.05^a^	21.00 ± 0.05^c^	36.00 ± 0.00
Fungi	Zones of inhibition (mm)			Ketoconazole
Aspergillus flavus RCMB 002002	12.00 ± 0.03^b^	11.00 ± 0.03^c^	13.00 ± 0.01^a^	16.00 ± 0.00
Penicillium italicum RCMB 001018	13.00 ± 0.02^b^	12.00 ± 0.02^c^	15.00 ± 0.03^a^	18.00 ± 0.00

The inhibition zone greater or equal to 6 mm was selected

Means with different superscript small letters in the same row indicate significant differences

Treatment 1: milk-fermented with *L. acidophilus* culture; treatment 2: milk-fermented with *L. acidophilus* and viable *S. boulardii* cultures; Treatment 3: milk-fermented with *L. acidophilus* and attenuated *S. boulardii* cultures

RCMB: Regional Center for Mycology and Biotechnology, Faculty of Pharmacy, Al-Azhar Univ., Cairo, Egypt

**Table 4:** Organic acids content (µg/mL) produced by *L. acidophilus* La-5 and/or *S. boulardii* 002Y018 in inoculated sterilized skim milk at 37 and 28 °C/24 h

Organic acids	*L. acidophilus*	*L. acidophilus *and *S. boulardii*	*S. boulardii*
Oxalic	76.270	108.03	6.18
Formic	536.75	519.98	232.37
Lactic	4190.96	5016.47	377.76
Acetic	243.15	298.04	57.92
Citric	879.53	848.01	967.21
Succinic	57.12	18.09	10.41
Propionic	877.14	2019.79	102.52

### Antidiabetic Activity

Alpha-amylase and α-glucosidase are the main enzymes are responsible for carbohydrates hydrolysis and elevated blood glucose where they promote carbohydrates breakdown and absorption. Thence, the inhibition of these enzymes leads to decrease post prandial glucose and control type 2 diabetes [26]. The ability of fermented milk treatments to inhibit α-amylase and α-glucosidase is listed in Table 5. All fermented milk treatments showed the ability to inhibit α-amylase and α-glucosidase with IC_50_ ranging from 8.88 to 23.93, and from 9.22 to 26.00 µg/mL, respectively. Compared to milk-fermented with *L. acidophilus* La-5 as a single culture (treatment 1), the addition of *S. boulardii* 002Y018 as a co-culture (treatment 2) led to significantly enhance the antidiabetic activity of fermented milk.

**Table 5:** IC_50_ values (µg/mL) of α-amylase and α-glucosidase Inhibition by fermented milk treatments on the first day of cold storage period

Treatments	Antidiabetic activity	
	α-amylase	α-glucosidase
Treatment 1	23.93 ± 1.12^a^	26.00 ± 1.56^a^
Treatment 2	8.88 ± 0.59^c^	9.22 ± 0.98^c^
Treatment 3	12.93 ± 0.76^b^	13.86 ± 0.51^b^
Acarbose	2.38 ± 0.10^d^	2.05 ± 0.11^d^
LSD	1.596	1.990

Acarbose was used as a positive control for the inhibition of α-amylase and α-glucosidase

Means with different superscript small letters indicate significant differences among fermented milk treatments for each parameter

Treatment 1: milk-fermented with *L. acidophilus* culture; treatment 2: milk-fermented with *L. acidophilus* and viable *S. boulardii* cultures; Treatment 3: milk-fermented with *L. acidophilus* and attenuated *S. boulardii* cultures

## Discussion

Few studies have conducted to investigate the use of viable or inactivated *S. boulardii* in the manufacturing of fermented dairy products. Therefore, this study focused on the ability of probiotic *S. boulardii* 002Y018 strain to grow in cow milk and hydrolyze proteins into various bioactive peptides in the presence of probiotic *L. acidophilus* La-5.

Using of *S. boulardii* 002Y018 in the viable or inactivated form did not affect the chemical composition of fermented milk. However, it positively affected on the titratable acidity, and this result proposed elevated lactose fermentation and/or metabolism in the probiotic dairy products during manufacturing, leading to a great production of organic acids and more acidic metabolites. Organic acids including lactic, acetic, formic, oxalic, propionic, and citric (Table 4) are the essential end metabolites of microbial fermentation that might differ in accordance with the probiotic species [27]. The increase in titratable acidity and decline in pH during the storage period might be interpreted to increase protein and lactose hydrolysis in the fermented milk, resulting in more releasing of acidic peptides and organic acids [28]. The production of exopolysaccharides by *S. boulardii* 002Y018 and their binding to milk proteins might be a cause for improving the viscosity and WHC of fermented milk [29, 30].

The competition between *S. boulardii* 002Y018 and *L. acidophilus* La-5 for energy, nitrogen sources, and low availability of the substances in the medium, particularly, through the last part of fermentation could decrease the viable count of *L. acidophilus* La-5 in the fermented milk of treatment 2, and this result was supported by Rasika et al. [31]. The available nutritional components in the environment including polysaccharides, short peptides, and free amino acids considerably affect the viability and growth of starter cultures. The inactivated *S. boulardii* 002Y018 provides vital fermentation environment substances, supporting *L. acidophilus* La-5 with nutritional demands for growth and maintaining its viability [17, 32]. Despite of the reduction in the viable counts of probiotic strains during the storage period, the total counts of single or co-cultures in all fermented milk treatments attained the recommended minimum quantity of 6.0 Log CFU/mL for probiotic microorganisms. Salvucci et al. [33] attributed the reduction in the viable counts of starter cultures during storage period to the increase in organic acid production particularly lactic acid and the deficiency in nutrients.

The addition of *S. boulardii* 002Y018 in the two forms enhanced the sensory attributes of fermented milk. Kang et al. [34] found that using of *Kluyveromyces marxianus* and *S. cerevisiae* as adjunct flavor cultures in milk-fermented with *St. thermophilus and L. bulgaricus* improved the sensory attributes. Zhang et al. [35] reported that some *Kluyveromyces marxianus* and *Saccharomyces cerevisiae* (commercial and wild type) strains generate organic acids, esters, and other flavor components through the fermentation process, gaining fermented dairy products a richer and more refreshing flavor, thus enhancing its evaluation.

The presence of *S. boulardii* 002Y018 in its two forms and the cold storage period revealed a positive impact on the protein hydrolysis in the fermented milk. In this respect, Rasika et al. [31] found that milk-fermented with *S. cerevisiae* K7 and *Lc. lactis* subsp. *lactis* NBRC 12,007 as a co-culture increased the proteolytic activity. Niamah [36] observed that the proteolytic activity of yogurt increased by the addition of *S. boulardii* from 200 to 250 µg/mL by 20%, while the rate of increase in the current study approximately 40%. Buts et al. [37] and Fakruddin et al. [38] reported that *S. boulardii* has proteolytic systems or enzymes that degrade milk proteins into peptides and free amino acids. Various scientific reports confirmed the increase of proteolysis in dairy products by the inoculation of *L. acidophilus* La-5 [21, 39]. Regarding the inactivated *S. boulardii* 002Y018 cells, using heat treatment for inactivation yeast cells increased releasing proteolytic enzymes and thus enhance the proteolytic activity [40].

As for ACE-I, and antioxidant activity, our findings exhibited that the proteases of *S. boulardii* 002Y018 either in the viable or inactivated form might contribute to release a wide range of ACE-I and antioxidant peptides in an excessive amount. The effect of co-culture might be probably due to microbial metabolic interactions leading to the C-terminal residue of peptides contained more proline that could be essentially responsible for the ACE-I activity [41, 42]. Abd El-Fattah et al. [21] reported that fermentation of milk with *L. acidophilus* La-5 led to release ACE-I, and DPPH radical scavenging peptides with activities of 36.8 and 68.8%, respectively. Sumny et al. [43] demonstrated that the whey fermentation with *S. boulardii* produced antioxidant peptides that are able to scavenge free radicals. Various studies illustrated that the antioxidant ability of peptides might be attributed to the existence of hydrophobic amino acids such as Val, Try, Phe, and Pro that bind to transition metals or free radical [44, 45]. Piame [30] pointed out certain strains of *S. boulardii* are potent to releasing carotenoids and polysaccharides that have antioxidant characteristics.

Using of *L. acidophilus* La-5 as a single culture or in a co-culture with *S. boulardii* 002Y018 for milk fermentation played a vital role in the growth inhibition of tested pathogenic bacteria and fungi. These data might be due to their releasing for organic acids including oxalic, formic, lactic, acetic, citric, succinic, and propionic in considerable values (Table 4; Fig. 4). Several studies reported that these organic acids possessed the ability to inhibit the growth of different pathogenic bacteria such as *Sal. typhimurium*, *L. monocytogenes*,* E. coli* 0157:H7, and *Staph. aureus* [46–49]. Offei et al. [50] found that some of *S. boulardii* has the ability to release high levels of acetic acid that possess an inhibitory effect on coliform bacteria. In addition, the antimicrobial activity of fermented milk might be due to the generation of antimicrobial peptides by *L. acidophilus* La-5 and *S. boulardii* 002Y018. Amiri et al. [51] found that *L. acidophilus* La-5 exhibited the ability to produce antimicrobial peptides that inhibit the growth of *S. aureus*,* L. monocytogenes*,* P. aeruginosa*,* E. coli*, and *S. enterica*. Pontier-Bres et al. [52] mentioned that *S. boulardii* can synthesize various proteases such as 54-kDa serine protease, which might degrade milk proteins into antimicrobial peptides. Promising investigations have exhibited different peptides produced via fermentation by yeast can inhibit the growth of pathogenic microorganisms through disrupting the wall constancy or binding to sites primary to the microorganism’s activity. These biopeptides are effective at pH 4–7 against various pathogenic microorganisms i.e. *E. coli*,* Klebsiella aerogenes*,* Staph. aureus*, and *Bacillus subtilis*. The mechanism of these peptides differs according to their charge where anionic peptides pass the wall membrane; compose pores resulting in uncontrolled interchange between extra- and intracellular compartments. However, cationic peptides react to the anionic constitutes of membranes leading to their laceration [53, 54]. Furthermore, our results showed that using of inactivated *S. boulardii* 002Y018 strain enhanced the antimicrobial activity of fermented milk and that might be attributed to the increase in the proteolysis, thus the increase in the antimicrobial peptides. Elshaghabee et al. [17] reported that the addition of heat-treated *Kluyveromyces lactis* NRRL Y-8279 at ratio of 3 and 5% to milk-fermented with ABT culture increased the proteolytic activity by 7.89 and 15.10%, respectively.

The addition of *S. boulardii* 002Y018 in the viable form promoted the inhibition of α-amylase and α-glucosidase (antidiabetic activity) compared to that of other fermented milk treatments. In this respect, Aquino et al. [55] reported that *S. boulardii* released biopeptides have the ability to inhibit dipeptidyl peptidase IV (DPP-IV) and α-glucosidase related to glucose metabolism. Ives et al. [56] and Abildinova et al. [57] mentioned that acetic and succinic acids that produced by *S. boulardii* play a crucial role in regulating lipids and carbohydrates metabolism. Acetic acid enhances insulin sensitivity and regulates gene expression related to glucose metabolism. Various scientific research interpreted the antidiabetic activity of fermented milk products might be attributed to production of different metabolites such as vitamins, lactate, and exopolysaccharides by LAB particularly *Lactobacillus* species [58, 59]. In addition, Ayyash et al. [25] and Elkashef et al. [60] indicated that milk-fermented by *Lactobacillus* species produce bioactive peptides inhibiting α-glucosidase and α-amylase. Castañeda-Pérez et al. [61] and Famuwagun et al. [62] clarified that small peptides had the strongest inhibitory activity against α-glucosidase and α-amylase, and they attributed that to the release of electrons or the existence of some amino acid residues, which react to the active or catalytic sites of the enzyme, thus inhibit enzyme activity.

**Fig. 5:**
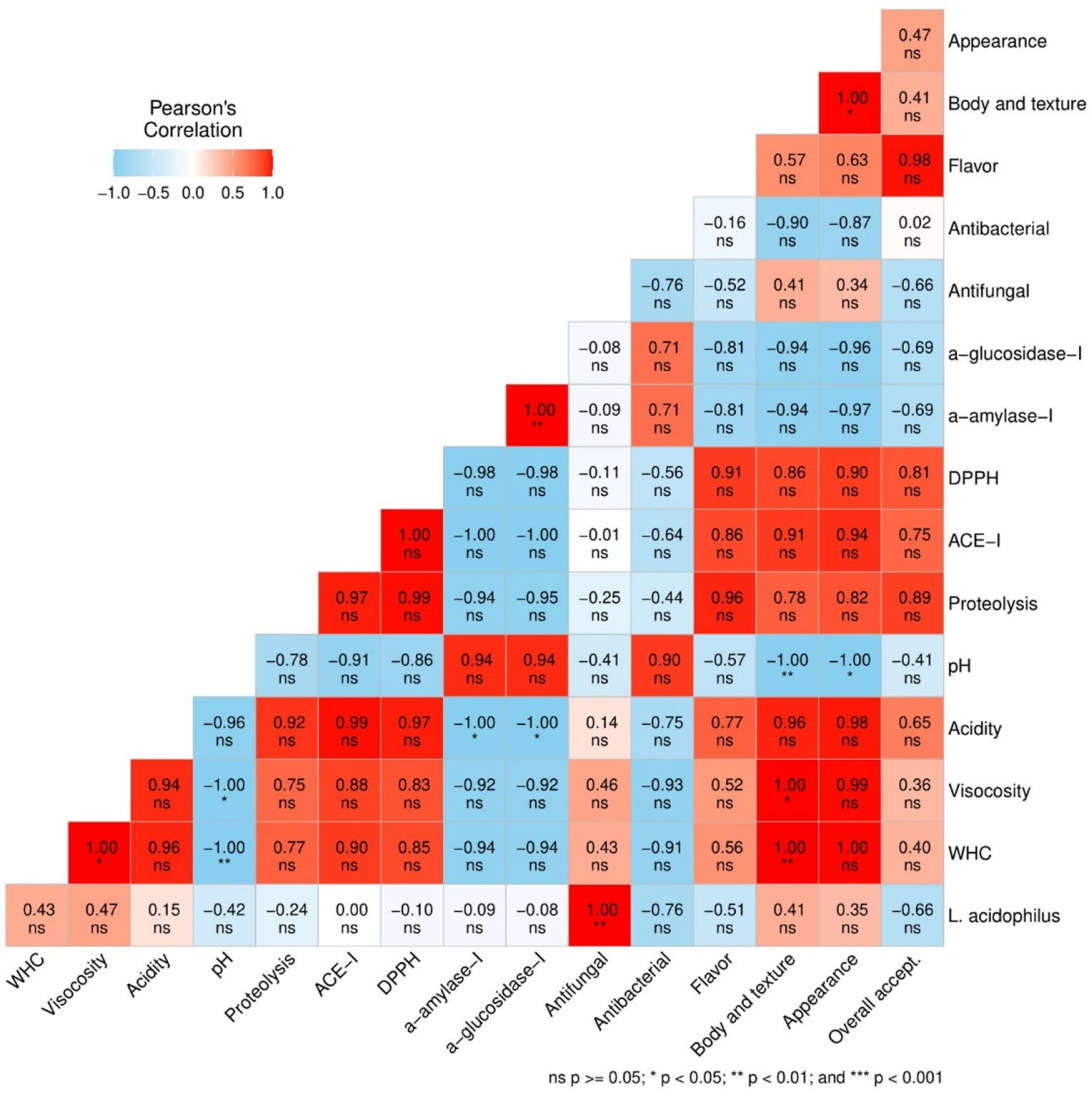
Heatmap plot illustrating the relationships matrix among the tested characteristics of various fermented milk treatments. Positive values indicate to positive relationships among two variables. The significance (*) points out the impact of fermented milk treatments on the relationships among variables

Depending on the tested measurements related to technological and functional characteristics of various fermented milk treatments, the relationships among the tested variables and the influence of probiotic strains on these relationships were assessed as demonstrated in heatmap results visualization plot (Fig. 5). A variation in the color from red to blue points out a decline in the relationship among different tested variables. It was observed a good relationship among proteolytic activity, and ACE-I or antioxidant activity. This result aligns with the results obtained by Abd El-Fattah et al. [18]. In addition, there were positive relationships between sensory and physical properties (viscosity and water-holding capacity). Otherwise, the heatmap plot illustrates that fermented milk treatments (probiotic strains) showed considerable impacts on the relationships between physical and sensory attributes as well as between proteolysis and antifungal activity or α-amylase inhibition. These findings indicate that the protease enzymes produced by *L. acidophilus* La-5 or *S. boulardii* 002Y018 could generate biopeptides enable to inhibit the growth of fungi and α-amylase enzyme [30]. Moreover, our findings pointed out the potential application of *S. boulardii* 002Y018 to produce fermented dairy products without a negative influence on the sensory attributes.

## Conclusion

The present work was planned to investigate the application of probiotic *Saccharomyces boulardii* 002Y018 strain in the viable or inactivated form for manufacturing of milk-fermented with probiotic *L. acidophilus* La-5 culture. The results exhibited that the using of *S. boulardii* 002Y018 in its two forms did not negatively affect the technological and sensory characteristics of fermented milk. The addition of *S. boulardii* 002Y018 in the inactivated form improved the growth of *L. acidophilus* La-5 culture. Furthermore, the inoculation of fermented milk with *S. boulardii* 002Y018 had a great effect on the functional properties including proteolytic, ACE-I, DPPH radical scavenging, antimicrobial, and antidiabetic activities. Consequently, our promising findings indicated that it could be applied probiotic *S. boulardii* 002Y018 strain in the viable or inactivated form to manufacturing of commercial fermented dairy products with great functional properties and without negative effect on the technological and sensory attributes.

## Data Availability

All results obtained regarding this work are involved in this published article.
